# CNT Enabled Co-braided Smart Fabrics: A New Route for Non-invasive, Highly Sensitive & Large-area Monitoring of Composites

**DOI:** 10.1038/srep44056

**Published:** 2017-03-08

**Authors:** Sida Luo, Yong Wang, Guantao Wang, Kan Wang, Zhibin Wang, Chuck Zhang, Ben Wang, Yun Luo, Liuhe Li, Tao Liu

**Affiliations:** 1 Beihang University, School of Mechanical Engineering & Automation, International Research Institute for Multidisciplinary Science, Beijing, 100191, China; 2 Florida State University, High Performance Materials Institute, Tallahassee, 32310, USA; 3 China University of Geosciences, Center of Safety Research, Beijing, 100083, China; 4 Georgia Institute of Technology, H. Milton Stewart School of Industrial and Systems Engineering, Atlanta, 30332, USA; 5 Stanford University, School of Medicine, Stanford, 94305, USA

## Abstract

The next-generation of hierarchical composites needs to have built-in functionality to continually monitor and diagnose their own health states. This paper includes a novel strategy for *in-situ* monitoring the processing stages of composites by co-braiding CNT-enabled fiber sensors into the reinforcing fiber fabrics. This would present a tremendous improvement over the present methods that excessively focus on detecting mechanical deformations and cracks. The CNT enabled smart fabrics, fabricated by a cost-effective and scalable method, are highly sensitive to monitor and quantify various events of composite processing including resin infusion, onset of crosslinking, gel time, degree and rate of curing. By varying curing temperature and resin formulation, the clear trends derived from the systematic study confirm the reliability and accuracy of the method, which is further verified by rheological and DSC tests. More importantly, upon wisely configuring the smart fabrics with a scalable sensor network, localized processing information of composites can be achieved in real time. In addition, the smart fabrics that are readily and non-invasively integrated into composites can provide life-long structural health monitoring of the composites, including detection of deformations and cracks.

Fiber-reinforced polymeric composites (FRPs) are remarkably important as structural materials for a wide variety of applications such as commercial & battle aircrafts, jet engine, wind turbine, gas and oil transmission pipelines etc. Since the last decade, substantial attention has been paid for the research and development of next generation of FRPs with built-in multifunctionality for self-sensing, identifying, quantifying and deciding their own health states^1^,^2^. In one aspect, different approaches were attempted to impart the FRPs with capabilities for structural health monitoring (SHM) during different stages of their life cycle^3^,^4^. For examples, SMARTweave^5^, optical fiber^6^,^7^, time domain reflectometry^8^, thermometer^9^, ultrasonic^10^, and pressure transducers^11^ have been used in the manufacturing stage of FRPs for resin infusion and curing monitoring. Similarly, optical fiber^12^, eddy-current^13^, piezoelectric^14^ and magnetostrictive sensors^15^ were used in the service stage of FRPs for deformation and crack detections.

As compared to the above mentioned traditional methods, carbon nanotubes (CNTs) enabled *in-situ* SHM technology has attracted a considerable amount of attention by virtue of their excellent mechanical robustness, non-invasive embeddability and conformability, light weight, low cost in fabrication and implementation, and remarkably high piezoresistive sensitivity^16^,^17^. Different types of CNT sensors have been explored, e.g., by bridging CNTs onto fiber and matrix interface as 1D sensors^18^,^19^, by depositing CNTs to formats of thin films or buckypapers as 2D sensors^20^,^21^,^22^,^23^ or by distributing CNTs directly in the resin matrix to form 3D sensors^24^,^25^. In most cases, the working principle of CNT sensors relies on the tunneling resistance change of the percolated CNT network upon external stimulus to impart the sensors with the capability for monitoring different mechanical deformations, cracks and failure modes in hierarchical composites^26^,^27^. In addition to monitoring the health state of FRPs in their service stage, it is equally important to be able to *in-situ* and in-line monitor the resin infiltration and curing kinetics in manufacturing process of composites. Because of the complicated fibrous preforms, resin infiltration is always non-uniform and hard to predict. This may introduce problematic flow defects such as resin-starved (dry spots) and resin-rich areas^28^. In addition, the curing kinetics is also susceptible to variations of the processing conditions. These mentioned manufacturing issues, in return, would introduce part-to-part variations and negative effects on macroscopic properties of the final composites, if it is lack of in-line monitoring approaches. Aiming to resolve these issues, Zhang *et al*. used electrophoretic method to fabricate CNT coated glass fiber for probing the curing process of epoxy resin^29^. By taking advantage of the unique porous structure of CNT and graphitic nanoplatelet (GNP) thin films, our group invented CNT and GNP thin film based fiber sensors for *in-situ* monitoring the manufacturing process of fiberglass prepreg laminates^30^,^31^.

Given the progress being made, however, there is still lack of a highly sensitive, reliable and scalable method for large-area monitoring the manufacturing stage of hierarchical composites. To approach this ultimate goal and advance the above mentioned emerging technology, we report here a novel strategy in developing smart fabrics comprised of co-braidable, scalable and designable CNT fiber sensors, which can be fabricated through a cost-effective and high-efficient dip coating process. The smart fabrics can be used as one layer of the woven roving preforms. As such, it is able to be readily integrated into a composite structure through the well-developed vacuum assisted resin transfer molding (VARTM) technique. The smart fabrics could provide a precise way to monitor and quantify various events occurred during the composite manufacturing stage, including resin infusion, gelation as well as curing kinetics. This unique functionality has been firmly corroborated through off-line rheological and DSC studies of a series of composite samples processed under varied curing temperatures and resin formulations. The most distinctive feature of this smart fabric is the built-in multiple fiber sensors that can serve as a sensing network for large area monitoring. This allows for mapping the localized information of composites in real time and, is highly valued for the quality assurance of composite manufacturing. Lastly, the sensor network offered by the smart fabrics naturally exists in the composite laminate and is capable of strain mapping and detection of cracks in the service stage. Considering the non-invasiveness, robustness, and large-area deployment capabilities as well as its built-in dual functionalities for structural health monitoring of composites over its lifespan, we expect the high advantage of smart fabrics for enhancing safety, performance and reliability of future lightweight composites.

## Results and Discussion

### Fabrication, Integration and Characterization of Smart Fabrics

**Figure 1:**
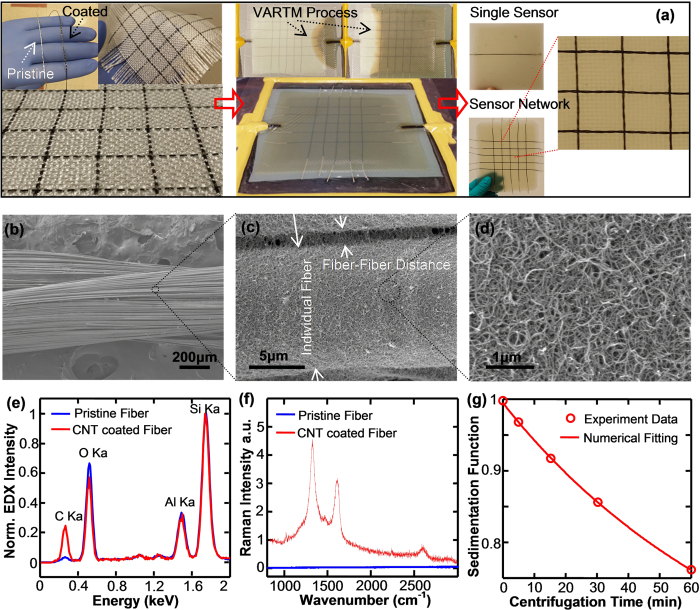
Assembly method and structural characterization of the smart fabrics. (**a**) Photographs of key steps in assembling the smart fabric composites, including fabrication of fiber sensors, formation of smart fabrics, and integration of composites; (**b–d**) SEM images of fiber sensors at different magnifications for examining the MWCNT packing structures on the fiber roving substrate; Comparison of (**e**) EDX and (**f**) Raman spectrum of a neat glass fiber roving and a MWCNT coated fiber sensor; (**g**) Experimentally determined and numerically fitted sedimentation function of MWCNT dispersion for calculating the bulk averaged length and diameter of nanotubes.

The smart fabric composites were prepared by three steps, including fabrication of CNT enabled fiber sensors, formation of smart fabrics, and composite manufacturing. First, a home-made roll-to-roll continuous process was established to fabricate the CNT enabled fiber sensors (see schematics of Figure S1, Supporting Information). This assembly was composed of a computer controlled motor (speed set at 1 cm/min) and a series of pulleys, which were used to pass a long fiberglass roving respectively through a multi-walled carbon nanotube (MWCNT) dispersion for thin film coating^32^, a deionized water bath for removing surfactant molecules (Triton-X-100), and a heating station for drying at ~200 °C. The smart fabrics were subsequently prepared by manually braiding the as-prepared fiber sensors into a woven roving cloth (Fig. 1a). Following the braiding process, they were stacked with other pristine fibrous plies to form dry composite preform, into which a mixture of vinyl ester resin and methyl ethyl ketone peroxide (MEKP) resin hardener were then immediately introduced through VARTM process operated in a vacuumed plastic bag to cause simultaneous in-plane and transverse resin wetting of the preform. During VARTM, the electric signal of fiber sensors embedded in the smart fabrics was simultaneously recorded to evaluate its capability for in-line monitoring of resin infusion and curing. After the manufacturing process, the smart fabrics existed in the composite laminates can serve for detecting various mechanical deformations and cracks. Additionally, it is scalable to a fiber sensor network to cover a large piece of composites. For demonstration purpose, the largest smart fabrics specimen we have made was ~300 cm^2^ with an incorporated 5 × 5 sensor array (Fig. 1a). Certainly, more sensors can be included to cover a larger composite.

To characterize the CNT structure coated on the fiber core, a variety of microscopy and spectroscopy methods have been used. First, based on visual inspection (Fig. 1a), the dark appearance of a fiber sensor as compared to the white color of a pristine fiber roving is a clear indication of a dense CNT film formed on the fiber surface through dip coating. In addition to visual inspection, SEM images provide detailed evidence of CNT coating as shown in Fig. 1b,c (low magnification) and 1d (high magnification). Albeit the small diameter of an individual fiberglass filament (15–20 μm), CNT nanoparticles were successfully coated on the fiber surface and assembled as rope/bundle entangled network morphologies. Similar morphologies can also be found on a large-area 2D substrate^33^,^34^. To further examine the successful coating of CNT on a glass fiber, we performed energy-dispersive X-ray (EDX) and Raman spectroscopy measurements on the fiber prior to and after the CNT coating. As normalized to the silicon peak intensity, EDX spectra (Fig. 1e) clearly show that the carbon peak is increased from 5.77 wt.% to 22.24 wt.% upon CNT coating. Similarly, the signature Raman features (Fig. 1f) of MWCNTs, i.e. G-band around 1600 cm^−1^, D-band around 1300 cm^−1^ and 2D-band around 2600 cm^−1^, confirm the coating process. To achieve a uniform CNT coating, one needs to bear in mind that a high quality CNT dispersion is a critical prerequisite. Thus, the quality of the CNT dispersion has been quantitatively analyzed using preparative ultracentrifuge method^35^,^36^,^37^. According to the experimentally determined sedimentation function and subsequent model fitting (Fig. 1g), we have calculated the averaged length (1845.2 nm) and diameter (3.74 nm) of the nanotube in dispersion. Based on weight measurements, the CNT content is determined to be less than 0.5 wt.% of the fiber sensor. With this piece of information and taking the market price of MWCNT (~$1 per gram), we estimated the cost of raw materials of the fiber sensor as low as ~$1.5 per 100 meters.

### Sensing Capability of Smart Fabrics for Process Monitoring

**Figure 2:**
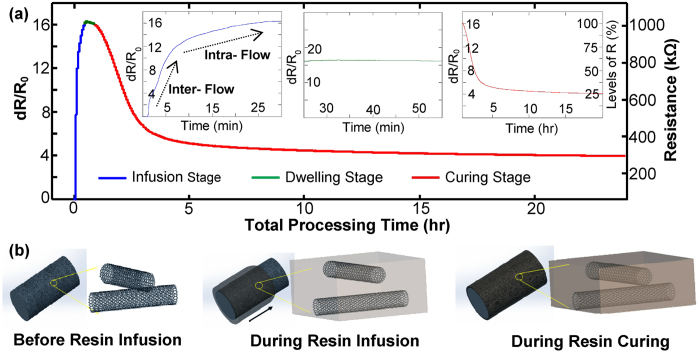
(**a**) Resin infusion and curing process of hierarchical composites registered by the real-time resistance change of the embedded smart fabric sensor. Infusion stage: 0–28 min (blue curve); Dwelling stage: 28 min – 55 min (green curve); Curing Stage: 55 min – 24 h (red curve); (**b**) Schematic diagrams of different packing structures of the CNT network in different stages of composite manufacturing.

Smart fabrics with a single fiber sensor have been studied principally to establish and quantify its sensing capability for monitoring resin infusion and curing. To specify, the fiber sensor with equal length in the fibrous ply was placed parallel to the direction of the resin infusion. To study its capability, real-time resistance signal of a representative smart fabric sensor was demonstrated in Fig. 2a. The total processing time of 24 h spans across the complete curing process, which is mainly categorized into three stages: 1) resin infusion stage including the time for injecting resin and hardener (1.25 wt.%) mixer into the pre-vacuumed plastic bag; 2) dwelling or pot-life stage when the resin has fully filled the bag but keeps a low viscosity; and 3) curing stage from the onset to the end of the polymer cross-linking reaction. Following the suggested curing protocols by the resin vendor, the curing temperature was isothermally controlled at 25 °C; and the bagging pressure was kept at 0.1 MPa.

By monitoring resistance change (*dR*/*R*_*0*_) of the single fiber sensor embedded in smart fabrics, as shown in Fig. 2a, a rapid increase of *dR*/*R*_*0*_ from 0 to ~11 (0–6 min) was accordingly observed and it was then smoothly merged into a milder increase (6–28 min) approaching to a stabilized value of ~16 (highlighted in blue). Subsequently, this maximum *dR*/*R*_*0*_ value was kept almost constant from 28 min to 55 min (highlighted in green). As the continuity of the process, a pronounced decrease of *dR*/*R*_*0*_ from ~16 to ~7 was initially observed (1 h – 3 h) and then gradually leveled off to a plateau value of ~4 toward the end of the process (3 h to 24 h, highlighted in red).

By correlating the resistance changes with physical states in composite manufacturing, it is interesting that the high repeatable trends of the sensor signal described above mimic closely to all the three stages of whole curing progression. First, as resin injecting and wetting the fibrous preform with fiber sensors, two types of flows compete with each other throughout the infusion process, namely, the inter- and intra-roving flow. Comparing to inter-roving distance ranging from hundreds of microns to several millimeters depending on density of the woven fabrics, intra-roving space is only micro- or nanoscale (clued in Fig. 1c). Thus the former speed to fill the voids among rovings is much faster than the latter speed to penetrate inside rovings. Following this line of thought, the different incremental rate of *dR*/*R*_*0*_ before and after the inflection point can be explained. For the first 6 min, the inter-roving flow dominates and it allows the resin molecules to wet the MWCNT network deposited on outer surface of the fiber roving to result in expansion and even breakage of tube/tube contacts, which causes the significant resistance increase (indicated by schematics of Fig. 2b). From 6 min to 28 min, the intra-roving flow dominates. Comparing to the first 6 min, it slowly but continuously penetrates/infiltrates the fiber roving to further disrupt MWCNT network. The hypothesis can be indirectly proved by video-taping of the resin flow (Figure S2, Supporting Information). It indicates after 6 min and 10s, the resin fills the bag. However, the detailed intra-fiber flow cannot be revealed. This reversely reflects the unique capability of smart fabrics such as for detecting detailed resin flow and tracking the resin flow front.

In addition to the infusion stage, we attribute the rest of the sensor signal to physical/chemical changes in the cross-linking reaction and the concomitant variations of system viscosity, as well as the development of matrix shrinkage caused by phase transformation of gelation and vitrification. Under low levels of cross-linking, the resin molecules retain low viscosity and do not disrupt the equilibrium state of the vacuumed system. As a result, the constant value of *dR*/*R*_*0*_ from 28 min to 55 min was observed. Continuing with the curing process, sufficiently high levels of cross-linking density would cause a drastic increase of the system viscosity and volumetric shrinkage of the resin. Consequently, the MWCNT network with infiltrated resin shrinks accordingly to cause its conductive paths closing together to have higher packing density, which is illustrated in the right scheme of Fig. 2b. Thus, the substantial decrease of *dR*/*R*_*0*_ from ~16 to ~4 was observed when the processing time ranges from 1 h to 24 h. To further corroborate the argument, we compared the CNT coated fibers to carbon fibers as another type of embedded roving sensors. The results demonstrated in Figure S3 (Supporting Information) indicate that the sensitivity of the smart fabric co-braided with CNT fibers is at least two orders of magnitude higher. With the rationale of carbon fibers with more densely packed graphitic structures, it provides clear evidence that the loosely packed CNT network can be interrupted much easier by physical/chemical changes of the resin. It is also important to note that due to exothermal reaction, the intrinsic transport property of the MWCNT network and thermal expansion of the laminates could contribute to resistance change in the curing stage. However, due to the relatively small temperature coefficient of MWCNT network (−0.137% K^−1^)^38^ and thermal expansion coefficient of fiberglass composites (~20 ppm/°C)^30^, these two effects are negligible as compared to the cross-linking effect.

**Figure 3:**
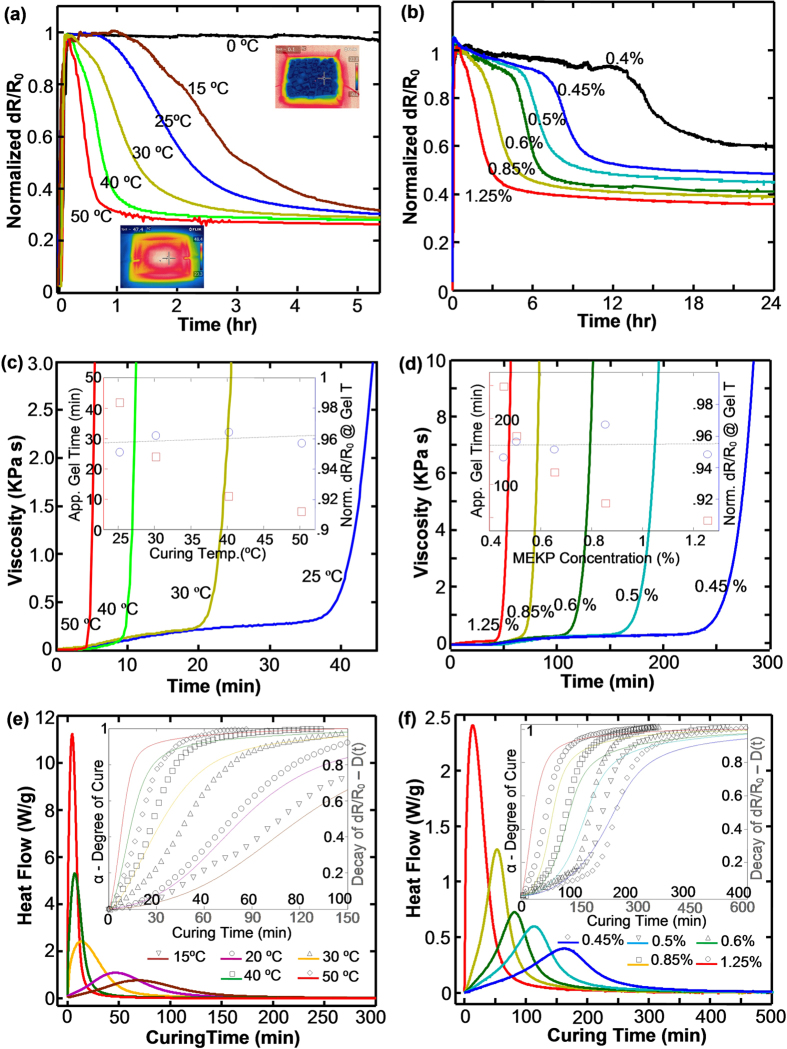
Process monitoring of composites using smart fabric, rheological and DSC methods. The three methods were respectively used for monitoring resistance change (**a,b**), viscosity (**c,d**) and heat flow (**e,f**) of two series of samples under various curing temperatures (**a,c,e**) and MEKP concentrations (**b,d,f**). Inset plots of (**c**) and (**d**) compares the rheometer determined gel time and the smart fabric sensor measured resistance change at the same gel time; Inset plots of (**e**) and (**f**) compares the DSC determined degree of cure and the smart fabric sensor measured decay of resistance change as a function of time.

To better understand the correlation between the sensor signal and cross-linking reaction of the polyester resin, we further argued that the dynamic resistance decay shown in Fig. 2a has a strong connection with the resin curing kinetics, such as gel time/point, onset and end of cure, percentage and rate of curing. To verify this hypothesis, we systematically investigated the effect of curing temperature and resin formulation by comparing the results from three different techniques, namely, smart fabric sensor, rheometer and differential scanning calorimetry (DSC). First, Fig. 3a,b respectively show the real-time signals of two series of smart fabric sensors for monitoring the curing process under varied curing temperatures and MEKP concentrations. To better convey the data, the resistance change (*dR*/*R*_*0*_) was normalized to its maximum value. By keeping the MEKP at 1.25 wt.%, Fig. 3a presents a clear trend that for a given resin formulation, the higher the curing temperature, the faster the decay of the sensor signal with respect to the processing time. The inset thermal images in Fig. 3a reveal the strategy for controlling curing temperature using ice water or heating stage (More details are shown in Figure S4, Supporting Information). As a representative example of 0 °C, *dR*/*R*_*0*_ keeps staying high after ramped up. This strongly indicates that the resin molecules hardly crosslink under this extreme condition. In addition, as increasing the controlled temperature from 15 °C to 50 °C, the dwelling duration that maintains the highest *dR*/*R*_*0*_ substantially decreased from ~70 min to ~6 min. For another instance, the elapsed time for normalized *dR*/*R*_*0*_ decayed from 1 to 0.5 was decreased from ~120 min (15 °C) to ~25 min (50 °C). Similar trends were also observed by varying the MEKP concentration from 0.4 wt.% to 1.25 wt.% while keeping the whole curing process at room temperature (~25 °C). For safety considerations, 1.25 wt.% MEKP was set as the highest level as the vendor suggested. Again, as raising the MEKP concentration, the sensor signal decays more rapid. It is also interesting that there is a large discrepancy on the final stabilized *dR*/*R*_*0*_ as the resin formulation is varied. For example, this value drops from 0.59 to 0.33 as MEKP increases from 0.4 wt.% to 1.25 wt.%. This shows a large contrast when compared with the case in Fig. 3a, in which all *dR*/*R*_*0*_ values were stabilized to 0.3 ± 0.05. We argue that the curing temperature only modifies the crosslinking velocity but the resin formation determines not only the curing rate but also the final degree of polymerization.

To quantify the ability for unveiling key parameters of curing kinetics, we further correlated the results of smart fabric sensors with that of a rheometer and DSC. Figure 3c,d show the viscosity profile of two series of pure resin samples monitored by a parallel-plate rheometer with a temperature-controlling chamber. Instead of emphasizing the fact that cure temperature and resin formulation have a profound influence on the profile, we stress that all viscosity curves have a critical upturn moment before diverging toward infinity. And, this critical moment could be used to represent gel time. Then, by extracting the approximate gel time from each viscosity profile and matching it accordingly in the resistive curve, it is rather interesting that all the determined *dR*/*R*_*0*_ (normalized) is almost identical (~0.96). This performance is confirmed in both the temperature and the MEKP series, as respectively shown in insets of Fig. 3c,d. Thus, this critical moment when *dR*/*R*_*0*_ decays to 0.96 can be used as an indicator for representing gel time.

In addition to rheological results, DSC provides more detailed information of curing kinetics by measuring the quantitative heat flow as a function of time. Again, by changing the isothermal temperature or varying the MEKP concentration, Fig. 3e,f show the heat flow curves of various resin samples by defining the onset and end of cure as the time of extrapolated baseline and the moment when the signal leveled off. Based on DSC curves, the degree of cure (α) and the rate of reaction (dα/dt) as a function of time can be estimated according to:


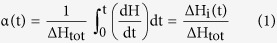



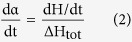


where ΔH_i_(t) is the partial heat of reaction at a certain time and ΔH_tot_ is the total heat of reaction. Calculated by Equation 1, inset of Fig. 3e,f presents the plot of α versus time at different isothermal temperatures or MEKP concentrations. It is clear that the higher the isothermal temperature or the MEKP concentration, the higher the fractional conversion of resin curing at a certain time. We found this behavior is highly related to the varying tendency of the smart fabric resistance. By selecting the resistive data in accordance with the onset and end of curing regulated by DSC, we defined the decay of resistance change as a function of curing time D(t) as:


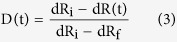


where dR_i_ and dR_f_ are the normalized resistance change at the onset and end of curing. As clearly demonstrated with the dotted curves in both insets of Fig. 3e,f, they preserve a strong correlation with DSC results for disclosing the quantitative degree of cure. Although the two sets of data are not perfectly coincident with each other, presumably caused by inaccurate temperature control using different heating sources and time mismatch considering different resin handling and transporting, the results of the smart fabric indeed restores the detailed features of the curing conversion in DSC curves. For instance, comparing to high temperature and MEKP samples with a fast ramping stage and a long leveling stage, the low temperature and MEKP samples show a much clearer three-stage behavior where the data is slowly ramped up followed by a high ramping stage and a slow conversion stage. By further comparing the rate of reaction (dα/dt) with the decay rate of resistance change (dD/dt), similar consequences happened (Figure S5, Supporting Information). Therefore, as compared with rheometer and DSC only adapted for off-line monitoring, the smart fabric is highly useful for in-line quantifying the resin curing in manufacturing stage of hierarchical composites.

### Large-area Monitoring of Smart Fabrics

**Figure 4:**
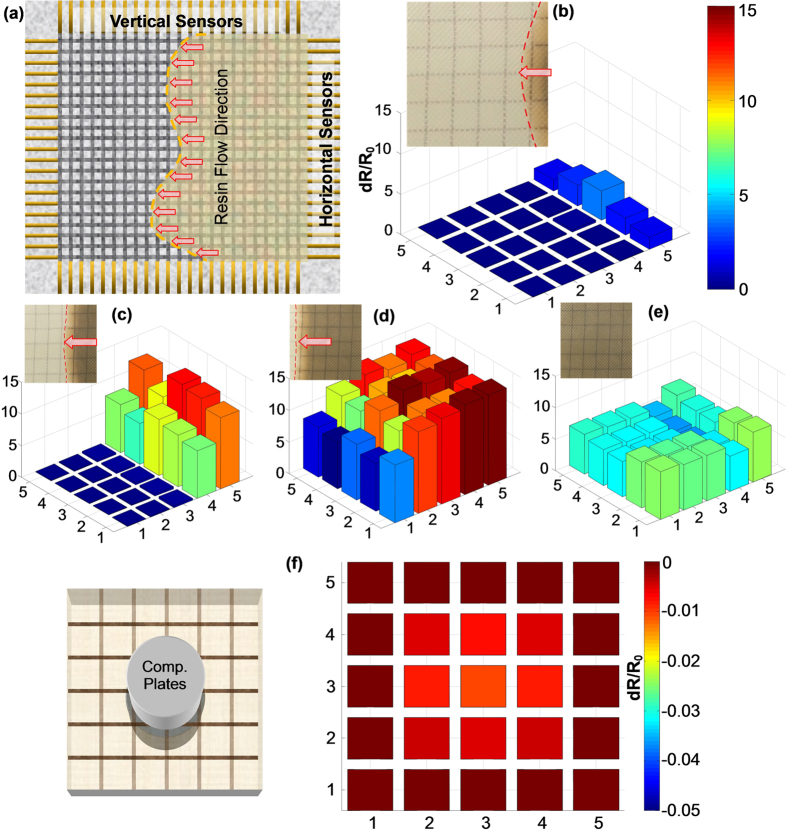
Versatile SHM capabilities of smart fabrics with scalable sensor network. (**a**) Schematic diagram of the smart fabric including multiple horizontal and vertical sensors for monitoring local information of the real-time resin infusion and curing process; (**b**–**e**) Distribution of the resistance change at every local points under different moments of resin infusion and curing by an embedded 5 × 5 sensor array; (**f**) Schematic diagram and experimental results of a fully cured laminate incorporating the same smart fabric for monitoring distribution of compression forces.

The smart fabric with a single sensor introduced in the previous section can be easily scaled up to a large sensing network by co-braiding multiple fiber sensors to monitor every local spot of composites. As schematically demonstrated in Fig. 4a and Figure S6 (Supporting Information), the aligned horizontal and vertical fiber sensors arranged in separate layers (not contacted by others) cover the whole piece of composites. Thus for a n × m sensor network with “n” horizontal fiber sensors (labeled as *Hi* from “*H1*” to “*Hn*”) and “m” vertical fiber sensors (labeled as *Vj* from “*V1*” to “*Vm*”), each sensor monitors the whole resistance change (*R*_*Hi*_ stands for resistance change of the *i*th horizontal sensor and *R*_*Vj*_ stands for resistance change of the *j*th vertical sensor) of the line region it covers during the whole process of resin infusion and curing. To extract the information of resin changes at the location defined by the cross point of *Hi* and *Vj* sensors, we defined *Rij* as the local resistance change of the *i*th horizontal sensor crossed by the *j*th vertical sensor. Thus, we formulated a model to describe each *Rij* by proportionally allocating *R*_*Hi*_ based on the ratios of all *R*_*Vj*_, as seen in Equation 4. One needs to notice that *Rij* is nothing but a technically defined quantity, which is *R*_*Hi*_ weighed by a factor. The approximation symbol (≈) is used because polynomial terms in the model are omitted but this would not alter the basic trend of resistance changes.


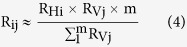


By simultaneously monitoring every horizontal and vertical fiber sensor in the smart fabric, Fig. 4b to e show the *dR*/*R*_*0*_ distribution at four representative moments of the resin process with a 5 × 5 sensor array. The optical image in Fig. 4b indicates that the curved resin head was just crossing the rightest vertical sensor line. At this critical moment, the resistance change of every local point was allocated based on Equation 4. Displaying each resistive value by a three-dimensional bar plot with a color scale, it is amazing that the shape of *dR*/*R*_*0*_ distribution has faithfully captured the position of the resin head. Clearly, the left 20 points remain unchanged because the resin has not started to wet those regions. In sharp contrast, *dR*/*R*_*0*_ of the right five points is strongly dependent upon their positions. To specify, the center point experiences the highest *dR*/*R*_*0*_. As deviating from the center location, the resin head gradually lags behind and the same is true for the resistance change of the corresponding points. Thus, we observed that R_55_ < R_45_ < R_35_ > R_25_ > R_15_. As the resin head continued to move to the left, Fig. 4c,d clearly showed that more and more regions were impacted by the resin transfer. A clear gradient of *dR*/*R*_*0*_ distribution was also found that the longer the region was suffered from the resin infusion, the higher the value of its resistance change. This was caused by the combination of inter-roving and intra-roving flow explained in the previous section. In addition to resin infusion, the *dR*/*R*_*0*_ distribution during the curing stage was also monitored. Figure 4e shows the corresponding results after three hours of processing. With similar blue-green colors, the magnitude of entire bars fell between ~5 and ~7, indicating all the local regions were subjected to similar degrees of curing. Based on its ability to map local areas with scalable size and density, we anticipate that the smart fabric technique presented will ensure full cure and no voids for manufacturing high quality composites.

In addition to monitoring the manufacturing process, the smart fabric sensors embedded in composite structures are readily to diagnose its health states after it is de-molded or debagged. Figure 4f gives an example to demonstrate the capability for capturing distribution of compression forces loaded on the composite laminate with the same 5 × 5 sensor array. The schematic picture shows that the cylindrical compression plates (50 mm in diameter) of the mechanical tester could only provide forces on the central part of the host composites. By applying a compression load of 450 N, the resistive response of each horizontal and vertical fiber sensor was converted to local *dR*/*R*_*0*_ values based on the same algorithm (Equation 4). The distribution results show that with negligible resistance changes on the peripheral area, clear *dR*/*R*_*0*_ changes near the central portion of composites were displayed in brighter colors, indicating its capability to detect local stresses and deformations. In addition to monitoring loadings and deformations, it is also highly valuable for detecting different failure modes of the composites, such as fiber/matrix delamination and crack initiation (Figure S7, Supporting Information). For instance, different modes of the composite laminate, i.e. elastic deformation (0–1.5%), initiation and development of micro-cracks or delaminations (1.5–5.5%) and catastrophic failure (>5.5%), were coincided with the substantially distinctive piezoresistive performance of the embedded sensor. Specifically, it has a gauge factor (GF) of 1.48/15.69/infinite value under elastic/crack/failure mode of composites, respectively.

## Conclusion

In conclusion, we demonstrated the robust and versatile sensory technology of smart fabric for diagnosing and evaluating the health states of polymeric composites from the manufacturing process to the service stage and finally to failure. By co-braiding MWCNT enabled fiber rovings into a fiberglass woven preform, we first demonstrated the use of the smart fabric sensor to provide *in situ* resin infusion and curing information during the vacuum assisted resin transfer molding (VARTM) process of composite manufacturing. Confirmed by rheological and DSC methods, the key processing parameters were quantitatively revealed with respect to resin flow, gelation and curing. We believe these findings are highly valuable and critical for quality assurance of the host composite structures. Then, the unique smart fabric sensor readily and noninvasively integrated into the laminate proved to be desirable for monitoring the strain and stress states, as well as for detecting the failures of the host structure. More importantly, the scalable size and adjustable sensing range of the smart fabric allows for covering a laminate of a comparatively large size and also suitable for monitoring the local information of resin processing and mechanical deformation. The multipurpose sensing capabilities in conjunction with their unique scalability and noninvasiveness make the smart fabric sensor highly valuable for life-long structural health monitoring of high-performance polymeric composites.

## Methods

### Fabrication and Characterization of Smart Fabric Sensors

CNT dispersion was prepared by dispersing 100 mg MWCNT (SWeNT^®^ SMW, bulk density of 0.22 g/cm3, averaged diameter of 5.5 nm, aspect ratio of 1000 and carbon content of >98%) in 200 mL of deionized water with 5 mL of nonionic surfactant triton X-100 (Sigma-Aldrich) in an ice bath using a Misonix 3000 probe sonicator (20 kHz). The sonicator was operated in a pulse model (on 10 s, off 10 s) with the power set at 45 W for 1 h. The length and diameter of CNTs in the resulting dispersion were characterized using preparative ultracentrifuge method (PUM) with the Optima MAX-XP ultracentrifuge (TLA-100.3, 30° fixed angle rotor, 13000 g-force, Beckman Coulter Inc.) and Delsa Nano C particle size analyzer (Beckman Coulter Inc.). The geometrical dimension of CNTs was also compared with images (Figure S8, Supporting Information) of MultiMode AFM (Veeco Instruments, Inc.). ScanAsyst and PeakForce Tapping probes (SCANASYST-AIR, Bruker) were used for AFM imaging. To fabricate smart fabrics, a fiberglass roving extracted from the woven fabrics (part # 223, Fibre Glast Developments Corp.) was used as the substrate of fiber sensors. As introduced in the previous section, the roll-to-roll coating process including a series of pulleys, a fixed heat gun (HG-301A, Master Appliance Corp.) and a computer-controlled stepper motor (Silverpak 17C, Lin Engineering Corps.) was used for converting the neat fiber roving into fiber sensors. They were then manually braided into the same woven fabric to form the smart fabric. Depending on different requirements, single or five parallel fiber sensors were incorporated in the smart fabric. The structures of CNT coating were examined by scanning electron microscopy (SEM), energy-dispersive X-ray (EDX) and Raman spectroscopy. SEM was performed with JEOL 7400 at 10 kV for examining the morphologies of the smart fabrics. The sample was sputter coated with gold prior to SEM imaging. The same instrument was used to acquire the EDX to determine the carbon content of sensors. A Renishaw inVia Raman microscope was used for collecting the Raman spectra of the smart fabric sensor with a 785 nm excitation laser at a power of 1 mW.

### Manufacturing of Composites Embedded with Smart Fabrics

The vacuum assisted resin transfer molding (VARTM) process was used to fabricate the composite laminate with embedded smart fabrics. Without specific notification, total of four layers of square shaped fabric (15 cm × 15 cm) were stacked into a multilayer composite preform. For single sensor experiments, the smart fabric layer was placed on top with the sensor parallel to the resin flow direction. For sensor array experiments, two smart fabric layers with five embedded sensors were respectively placed on top and at the bottom. The top (bottom) layer has the sensors parallel (vertical) to the resin flow direction. Copper electrodes were then connected to every two ends of fiber sensors. Subsequent to sensor integration and preform stacking, the fibrous stack was then placed in a vacuumed plastic bag to induce the resin infusion and curing process. The VARTM process was operated close to the standard atmospheric pressure (0.1 MPa) with a controlled temperature. Then the mixture of vinyl ester resin (part # 1110, Fibre Glast Developments Corp.) catalyzed by methyl ethyl ketone peroxide (MEKP-925, Norac, Inc.) with a certain mixing ratio was introduced to fill the vacuum bag. The total processing was isothermally maintained for 24 h. Subsequently, the composite laminate was debagged.

### Sensing Performance of Smart Fabrics for SHM of composites

To monitor the process of composite manufacturing, the resistance of embedded sensors was recorded by a Keithley 2401 Sourcemeter controlled by a homemade LabVIEW user interface during the whole curing process^39^,^40^. The curing temperature during each process was tested by a FLIR E40 thermal imaging camera. The rheological tests were performed by an ARES-LS3 rheometer (TA Instruments) with 25 mm parallel plate fixture. Oscillatory shear flow experiments with 5% strain and 1 Hz frequency were carried out under an isothermal cure temperature controlled in the furnace. The differential scanning calorimetry tests were performed by a Q-100 DSC (TA Instruments) by monitoring the heat flow under isothermally controlled temperature. The stretching and compression tests were performed using Shimadzu AGS-J micro test frame with a 5000N load cell. Based on tension-to-failure test, mechanical properties of the composites including tensile modulus (7.15 GPa) and tensile strength (259.2 MPa) were measured as referenced information.

## Additional Information

**How to cite this article:** Luo, S. *et al*. CNT Enabled Co-braided Smart Fabrics: A New Route for Non-invasive, Highly Sensitive & Large-area Monitoring of Composites. *Sci. Rep.* **7**, 44056; doi: 10.1038/srep44056 (2017).

**Publisher's note:** Springer Nature remains neutral with regard to jurisdictional claims in published maps and institutional affiliations.

## Supplementary Material

Supplementary Information
