# Supplementary material for: CNT Enabled Co-braided Smart Fabrics: A New Route for Non-invasive, Highly Sensitive & Large-area Monitoring of Composites
Source: Sci Rep. 2017 Mar 8;7:44056. doi: 10.1038/srep44056 (PMC5341057; doi:10.1038/srep44056)
Supplement: Supplementary Information [file srep44056-s1.pdf]

Supporting Information

CNT Enabled Co-braided Smart Fabrics: A New Route for Non-invasive, Highly Sensitive & Large-area Monitoring of Composites

Sida Luo[1,2,], Yong Wang[1], Guantao Wang[1,5], Kan Wang[3], Zhibin Wang[4], Chuck Zhang[3], Ben Wang[3], Yun Luo[5], Liuhe Li[1], Tao Liu[2,]**

[1]Beihang University, School of Mechanical Engineering & Automation, International Research Institute for Multidisciplinary Science, Beijing, 100191, China
[2]Florida State University, High Performance Materials Institute, Tallahassee, 32310, USA
[3]Georgia Institute of Technology, H. Milton Stewart School of Industrial and Systems Engineering, Atlanta, 30332, USA
[4]Stanford University, School of Medicine, Stanford, 94305, USA
[5]China University of Geosciences, Center of Safety Research, Beijing, 100083, China
*s.luo@buaa.edu.cn, tliu@fsu.edu

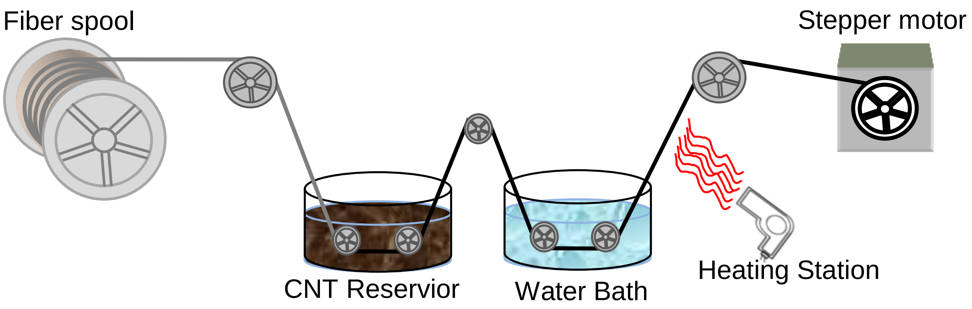

Figure S1. Schematic diagram of roll-to-roll assembly for continuous coating of MWCNTs on the fiberglass roving substrate.

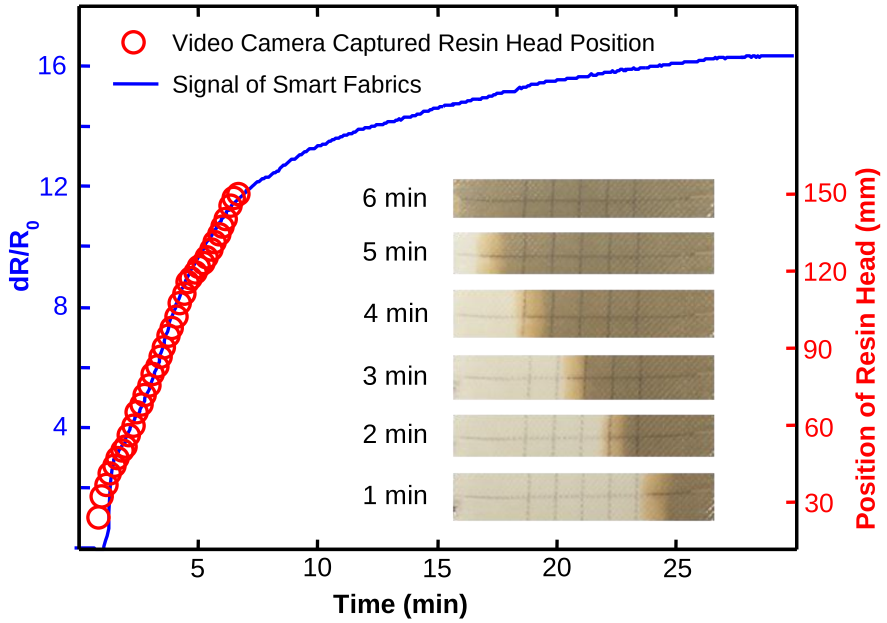

Figure S2. Real-time resistance change monitored by smart fabrics versus the real-time resin head positions captured by video-taping method. The camera could only record the first ~6 min of the resin flow indicating the resin has plausibly filled the bag. The resistive signal of smart fabrics could not only be coincident with the data of video camera to capture the resin head positions, but also reflect more detailed intra-roving flows after 6 min. The inset optical photographs are snapshots of the recorded video under varied resin infusion time

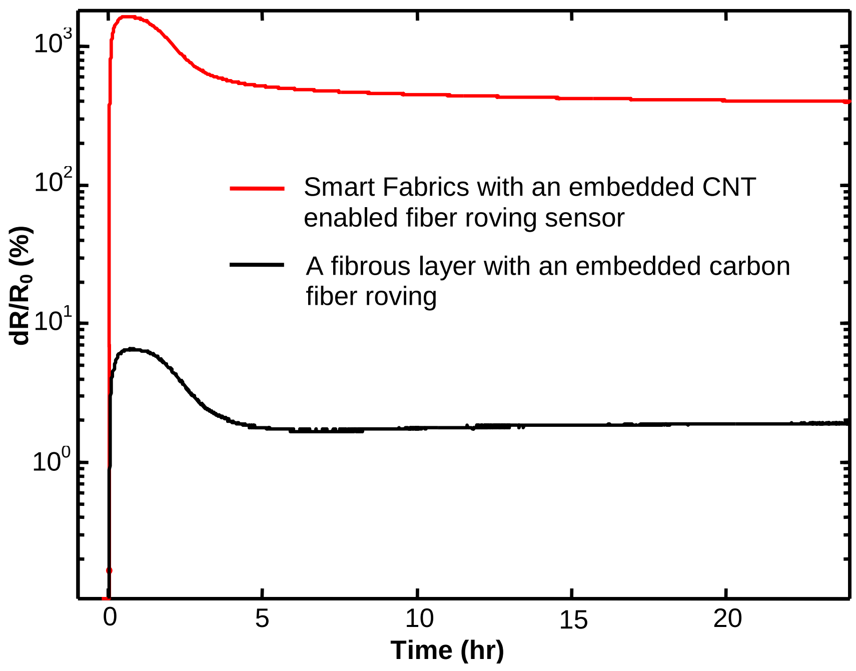

Figure S3. Real-time resistance change of a representative smart fabric sensor embedded with a single CNT enabled fiber roving sensor versus the same resistive signal of an embedded carbon fiber roving as a comparison between their capabilities for in situ curing monitoring of polymeric composites.

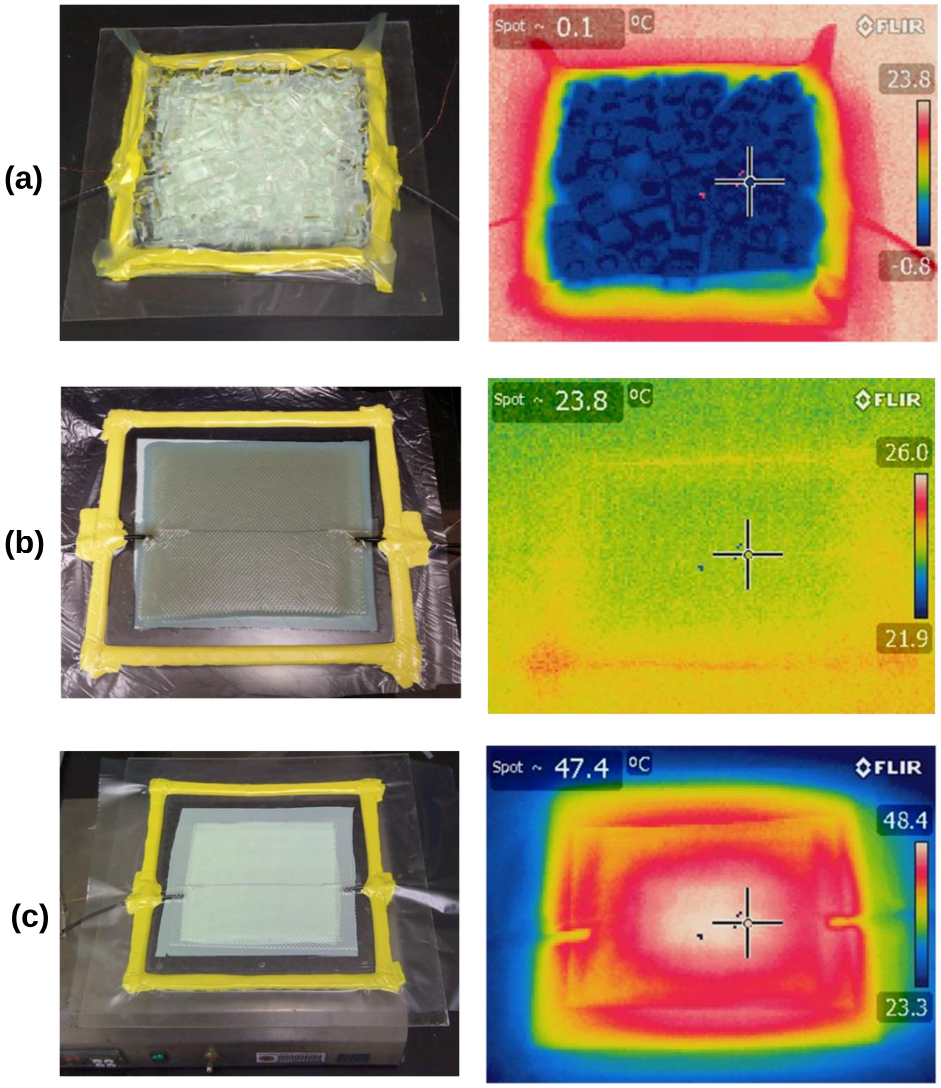

Figure S4. Representative optical and thermal images captured by a portable thermal imaging camera to reveal the temperature control strategies during the VARTM process isothermally controlled under (a) 0 ℃ using ice; (b) room temperature; and (c) 50 ℃ using a heating stage.

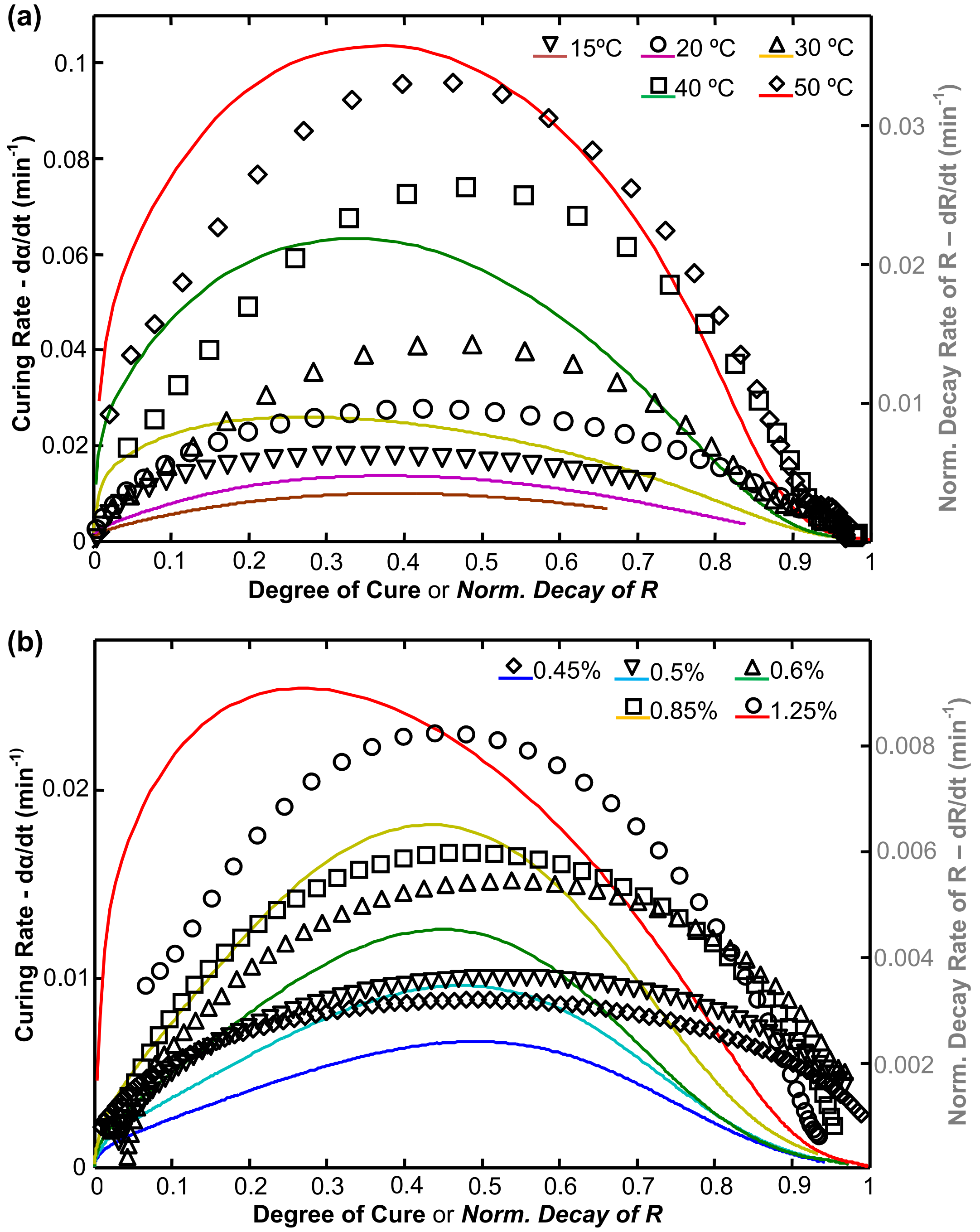

Figure S5. DSC determined curing rate (dα/dt) and smart fabric sensor tested normalized decay rate of resistance change (dR/dt) of series of laminate samples fabricated under (a) various curing temperature and (b) various MEKP concentrations.

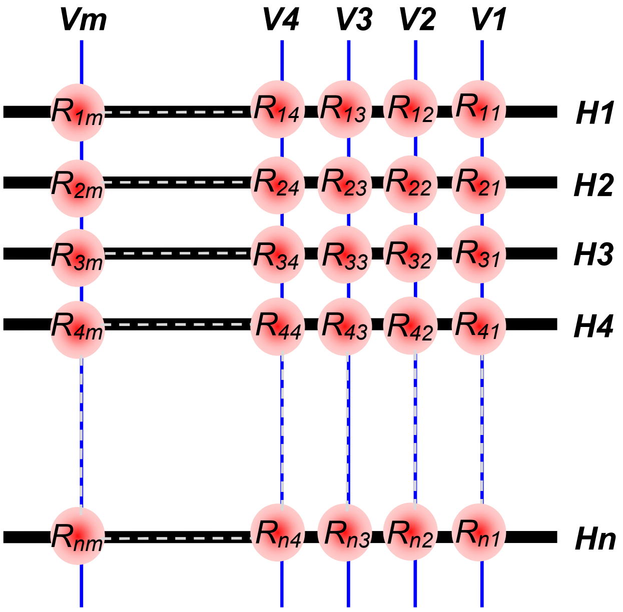

Figure S6. Schematic diagram of the smart composite fabrics co-braided with "n" horizontal fiber sensors (labeled as *Hi* from "*H1*" to "*Hn*") and "m" vertical fiber sensors (labeled as *Vj* from "*V1*" to "*Vm*"). *Rij* describes the local resistance change of the *i*th horizontal sensor covering the crossing area between *Hi* and *Vj*. To predict the *Rij* distribution with higher level of accuracy, it is necessary to incorporate another smart fabric layer with a series of co-braided 45 degree fiber sensors.

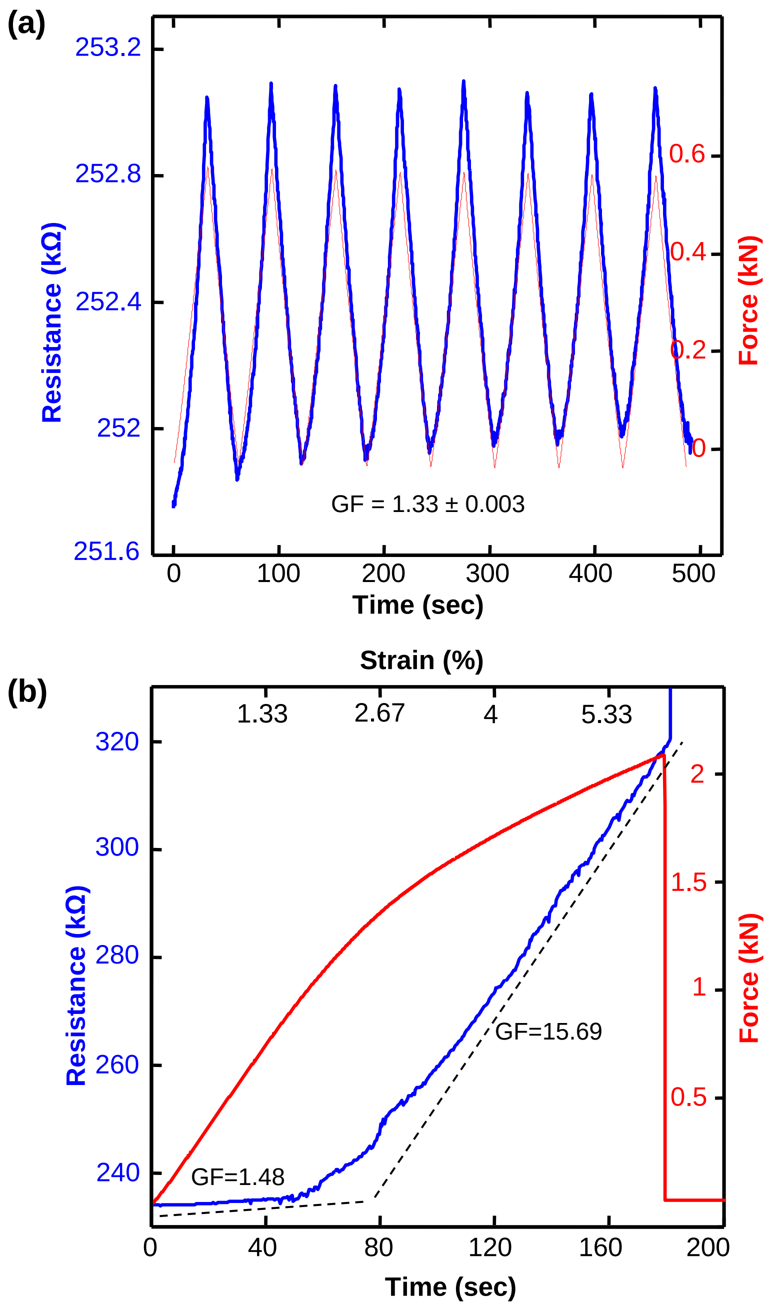

Figure S7. (a) Cyclic tensile test and (b) tension-to-failure test with simultaneous electrical measurements of a fully cured fiberglass composite embedded with a smart fabric sensor. The blue line is the sensor resistance response with respect to different forces (red line) applied to the matrix structure. Under small forces or deformations (strain < 0.35 %), the sensor shows a gauge factor of 1.33. Under tension-to-failure test, different modes of the composites have been revealed by distinct gauge sensitivity of the sensor, i.e., GF=1.48 which indicates the elastic deformation (0 – 1.5 %) of composites; GF=15.69 indicates the initiation and development of micro-cracks or delaminations (1.5 % - 5.5 %) of composites; GF=infinity which indicates the catastrophic failure (> 5.5 %) of composites.

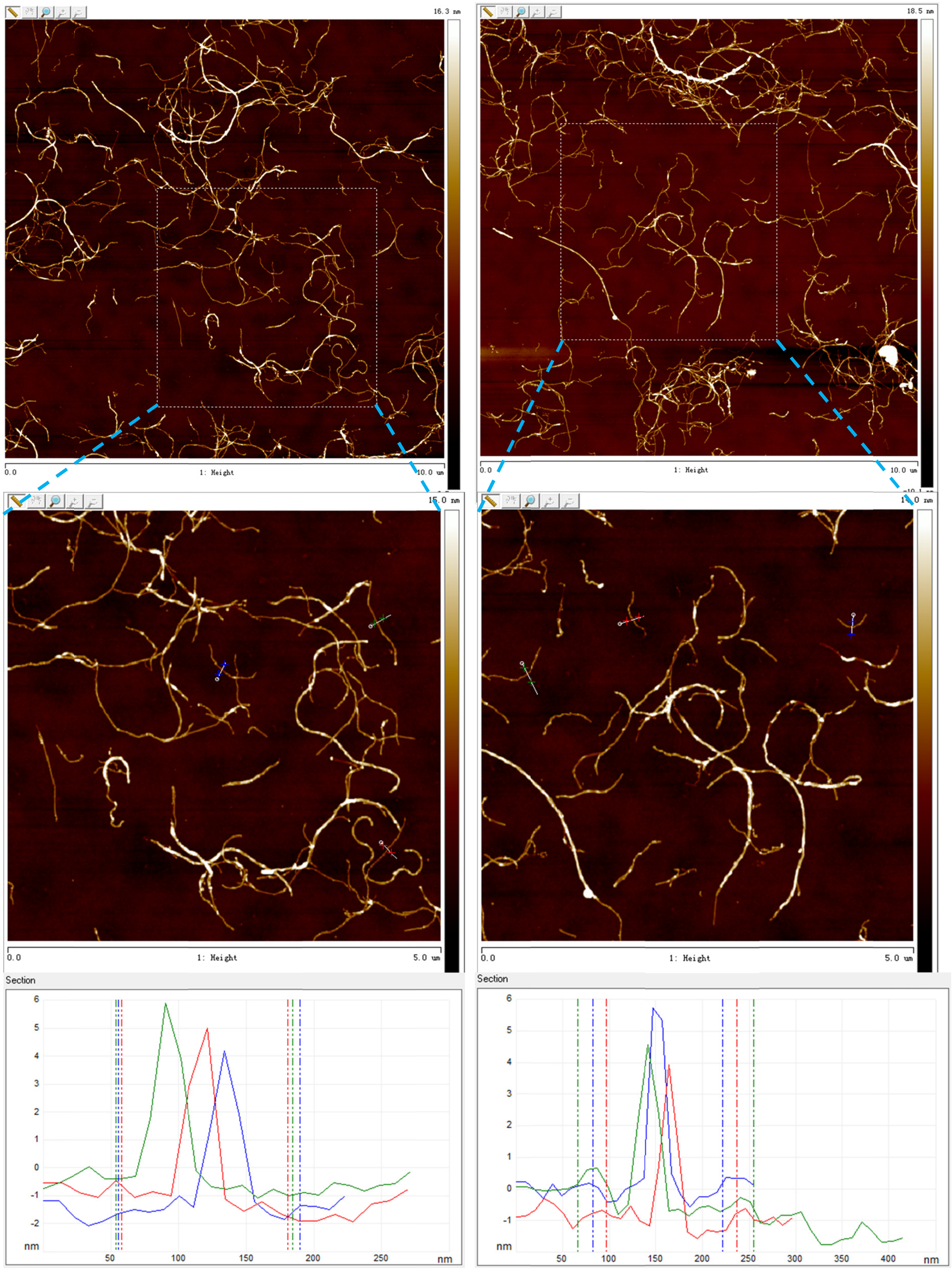

Figure S8. AFM images to measure geometrical dimensions of MWCNTs in a certain sonicated dispersion. The MultiMode AFM (Veeco Instruments, Inc.) was used to acquire images of CNTs under ambient conditions. ScanAsyst and PeakForce Tapping probes (SCANASYST-AIR, Bruker) with a triangular shaped cantilever and a rotated (symmetric) tip were used for AFM imaging. The sample was prepared by spin casting a diluted dispersion droplet on a silicon wafer (size ~ 1 cm × 1cm) at 2000 rpm. The sample was then baked in a vacuum oven at 180 °C overnight before undergoing AFM imaging.
